# Temperature

**Published:** 1890-03-15

**Authors:** 


### TEMPERATURE.

An authority on the subject observes: "The sources of animal heat must be chiefly sought for in medical processes, especially oxidation, which are constantly going on in the blood and tissues. To a minor degree various processes of a purely physical nature, such as friction, or the transformation into heat of other forms of energy themselves the outcome of chemical processes, have also a share in its production." In healthy men, as we all know, the temperature of the body measured in the axilla is about 98·6 deg., and this whether the climate or weather is hot or cold, radiation or evaporation coming more or less into play to secure this result. But during disease the temperature may rise to 108 deg. or more. This elevation of temperature has been usually attributed to altered or increased chemical process; to increase of oxidation in short. Most modern theories give a more prominent part to microbes in the blood, which in themselves, or by products of their own life-changes, only the changes they produce in the fluids of the body cause the febrile process or increase of temperature. But Dr. Pearse, of Plymouth, in an article headed 98·4 deg. in the "British Medical Journal" asks: "In a case of sudden hysterical pyrexia, when the temperature rapidly rises to 112 deg. and suddenly falls to normal, surely we shall not rest as an explanation on the word oxidation." Sudden rises and sudden falls of temperature are also often seen in children. In children the temperature sometimes increases rapidly, probably from stomach derangement, when there is nothing serious the matter, so that care is necessary when dealing with children not to form a hasty conclusion of some serious disease, simply because the thermometer indicates much heat of surface which often falls with rapidity. Again there is such a thing as local increase of temperature (not connected with any local inflammation). This local increase of temperature is sometimes seen in connection with so-called malarious disease. For example, a limb may become cold, hot, and sweat, passing through locally the phenomena of an ague attack. Now in the case of the hysterical increase of temperature, and in the rises afterwards mentioned, it is very easy to attribute such increases to "nervous influence." That mental influence does cause increased temperature is daily evidenced when a person from fright, or fear, or shame, or other cause, becomes "hot all over." But mental emotions do not much come into play when disease induces increased temperature. We should be glad if some one would explain "nervous influence" which is so glibly talked about. The question of temperature still requires some elucidation. A standard author (Ziempen) observes: "In the elevation of temperature which takes place during a paroxysm of fever, it is not merely a matter of increased production of heat and diminished loss of heat, but we have to do with a modification of, or disturbance of the power that regulates the heat of the body." This has given rise to the belief in the existence of a special centre presiding over the regulation of animal heat. Whether this is an excito-caloric or a moderating centre, or whether it possesses a double character we do not know. Neither has it been proved that there is any such special centre.

